# Crystal structure of 3-amino-2-propyl­quinazolin-4(3*H*)-one

**DOI:** 10.1107/S2056989015013134

**Published:** 2015-07-22

**Authors:** Gamal A. El-Hiti, Keith Smith, Amany S. Hegazy, Saud A. Alanazi, Benson M. Kariuki

**Affiliations:** a Cornea Research Chair, Department of Optometry, College of Applied Medical Sciences, King Saud University, PO Box 10219, Riyadh 11433, Saudi Arabia; b School of Chemistry, Cardiff University, Main Building, Park Place, Cardiff CF10 3AT, Wales

**Keywords:** crystal structure, quinazolin-4(3*H*)-one, hydrogen bonding, π–π overlap

## Abstract

In the title mol­ecule, C_11_H_13_N_3_O, the propyl group is almost perpendicular to the quinazolin-4(3*H*)-one mean plane, making a dihedral angle of 88.98 (9)°. In the crystal, mol­ecules related by an inversion centre are paired *via* π–π overlap, indicated by the short distances of 3.616 (5) and 3.619 (5) Å between the centroids of the aromatic rings of neighbouring mol­ecules. Inter­molecular N—H⋯N and N—H⋯O hydrogen bonds form *R*_6_^6^(30) rings and *C*(5) chains, respectively, generating a three-dimensional network. Weak C—H⋯O inter­actions are also observed.

## Related literature   

For biological applications of related compounds, see: Sasmal *et al.* (2012 ▸); Rohini *et al.* (2010 ▸); Chandregowda *et al.* (2009 ▸); Gupta *et al.* (2008 ▸); Alagarsamy *et al.* (2007 ▸). For the synthesis of substituted quinazolin-4(3*H*)-ones, see: Ma *et al.* (2013 ▸); Adib *et al.* (2012 ▸); Xu *et al.* (2012 ▸); Kumar *et al.* (2011 ▸). For modification of the quinazolin-4(3*H*)-one ring system *via* li­thia­tion, see: Smith *et al.* (2004 ▸, 1996 ▸, 1995 ▸). For the crystal structures for related compounds, see: El-Hiti *et al.* (2014 ▸); Yang *et al.* (2009 ▸); Coogan *et al.* (1999 ▸).
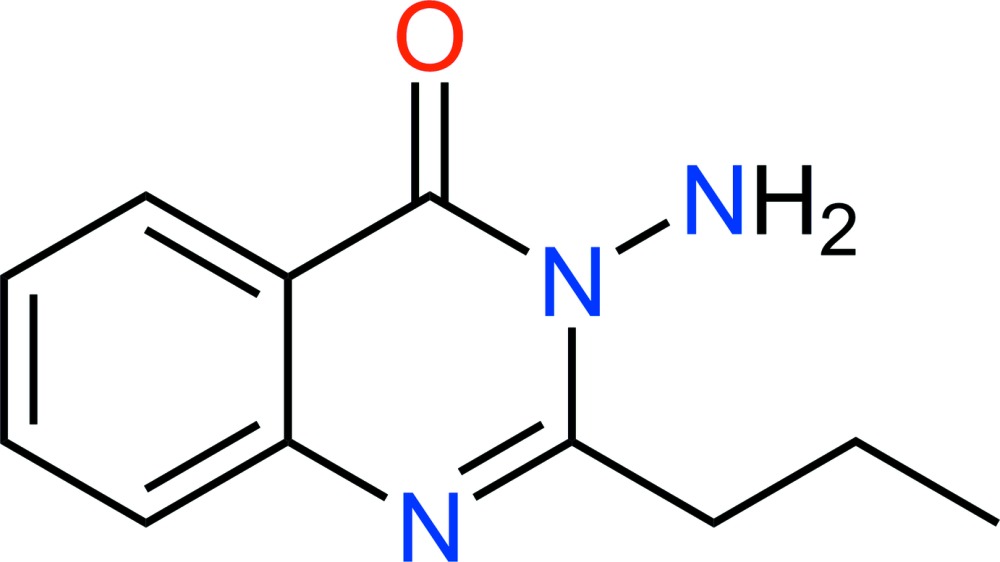


## Experimental   

### Crystal data  

C_11_H_13_N_3_O
*M* *_r_* = 203.24
Trigonal, 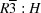

*a* = 24.1525 (5) Å
*c* = 9.6500 (2) Å
*V* = 4875.1 (2) Å^3^
*Z* = 18
Cu *K*α radiation
μ = 0.67 mm^−1^
*T* = 296 K
0.34 × 0.25 × 0.19 mm

### Data collection  

Agilent SuperNova Dual Source diffractometer with an Atlas detector
Absorption correction: multi-scan (*CrysAlis PRO*; Agilent, 2014 ▸) *T*_min_ = 0.975, *T*_max_ = 0.984
3734 measured reflections
2136 independent reflections
1913 reflections with *I* > 2σ(*I*)
*R*_int_ = 0.013

### Refinement  

*R*[*F* ^2^ > 2σ(*F* ^2^)] = 0.040
*wR*(*F* ^2^) = 0.120
*S* = 1.06
2136 reflections
146 parameters
H atoms treated by a mixture of independent and constrained refinement
Δρ_max_ = 0.17 e Å^−3^
Δρ_min_ = −0.15 e Å^−3^

### 

Data collection: *CrysAlis PRO* (Agilent, 2014 ▸); cell refinement: *CrysAlis PRO*; data reduction: *CrysAlis PRO*; program(s) used to solve structure: *SHELXS2013* (Sheldrick, 2008 ▸); program(s) used to refine structure: *SHELXL2013* (Sheldrick, 2015 ▸); molecular graphics: *ORTEP-3 for Windows* (Farrugia, 2012 ▸); software used to prepare material for publication: *WinGX* (Farrugia, 2012 ▸) and *CHEMDRAW Ultra* (Cambridge Soft, 2001 ▸).

## Supplementary Material

Crystal structure: contains datablock(s) I, New_Global_Publ_Block. DOI: 10.1107/S2056989015013134/cv5493sup1.cif

Structure factors: contains datablock(s) I. DOI: 10.1107/S2056989015013134/cv5493Isup2.hkl

Supporting information file. DOI: 10.1107/S2056989015013134/cv5493Isup3.cml

**Fig. 1.**
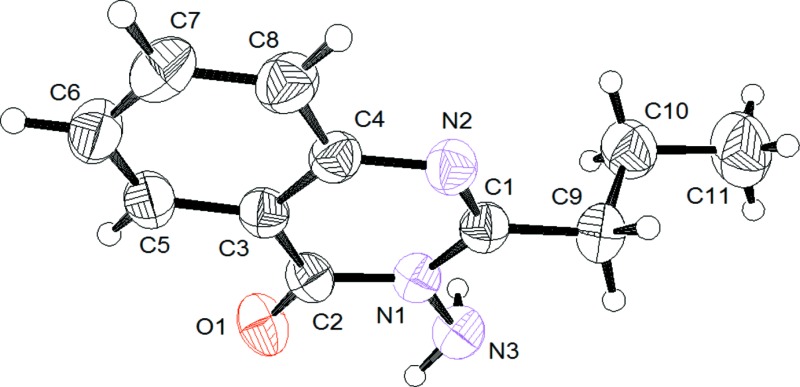
View of (I) showing the atom labels and 50% probability displacement ellipsoids.

**Fig. 2.**
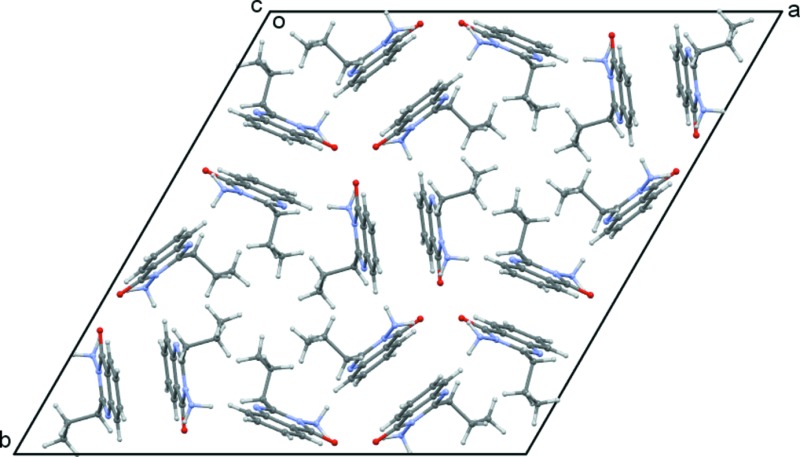
Crystal packing viewed along the c axis.

. DOI: 10.1107/S2056989015013134/cv5493fig1.tif
View of (I) showing the atom labels and 50% probability displacement ellipsoids.

c . DOI: 10.1107/S2056989015013134/cv5493fig2.tif
Crystal packing viewed along the *c* axis.

CCDC reference: 1411448

Additional supporting information:  crystallographic information; 3D view; checkCIF report

## Figures and Tables

**Table 1:** Hydrogen-bond geometry (, )

*D*H*A*	*D*H	H*A*	*D* *A*	*D*H*A*
N3H3*A*N2^i^	0.91(2)	2.16(2)	3.0677(17)	176.1(16)
N3H3*B*O1^ii^	0.87(2)	2.51(2)	3.0599(16)	122.0(15)
C5H5O1^iii^	0.93	2.44	3.3163(16)	157

Symmetry codes: (i) 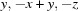; (ii) 
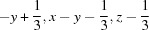
; (iii) 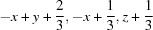.

## supplementary crystallographic information

### S1. Introduction

Quinazolines have a range of biological activities such as anti-cancer (Chandregowda *et al.*, 2009), anti-bacterial (Rohini *et al.*, 2010), anti-inflammatory (Alagarsamy *et al.*, 2007), anti-obesity (Sasmal *et al.*, 2012) and anti-spasm (Gupta *et al.*, 2008). Synthesis of quinazolin-4(3*H*)-ones involves use of various synthetic procedures. Recent examples involve reactions of 2-amino­benzo­nitrile with carbon dioxide in water (Ma *et al.*, 2013), 2-bromo­benzamides with formamide catalysed by CuI and 4-hy­droxy-l-proline (Xu *et al.*, 2012) and isatoic anhydride, benzyl halides and primary amines under mild Kornblum conditions (Adib *et al.*, 2012). 2-Alkyl-3-amino­quinazolin-4(3*H*)-ones can be obtained from reactions of 2-alkyl-4*H*-3,1-benzoxazin-4-ones with hydrazine hydrate (Kumar *et al.*, 2011). Li­thia­tion of 2-unsubstituted and 2-n-alkyl-3-acyl­amino­quinazolin-4(3*H*)-ones followed by reactions of the lithium reagents produced *in-situ* with electrophiles gave the corresponding substituted derivatives in high yields (Smith *et al.*, 2004, 1996, 1995). For the X-ray structures for related compounds, see: El-Hiti *et al.* (2014); Yang *et al.* (2009); Coogan *et al.* (1999).

### S2.1. Synthesis and crystallization

3-Amino-2-propyl­quinazolin-4(3*H*)-one was obtained in 82% yield by reaction of 2-propyl-4*H*-3,1-benzoxazin-4-one with excess hydrazine hydrate (three mole equivalents) in methanol under reflux conditions for 3 h (Kumar *et al.*, 2011). Crystallization from ethanol gave colourless crystals of the title compound. The NMR and mass spectral data for the title compound were identical with those reported (Kumar *et al.*, 2011).

### S2.2. Refinement

The amino hydrogen atoms were located in the difference Fourier map and refined freely. The rest of the H atoms were positioned geometrically and refined using a riding model with *U*_iso_(H) constrained to be 1.2 times *U*_eq_ for the atom it is bonded to except for methyl groups where it was 1.5 times with free rotation about the C—C bond.

### S3. Results and discussion

In the title compound (I) (Fig. 1), the propyl group is perpendicular to the quinazolin-4(3*H*)-one group with a dihedral angle of 88.98 (9)° between the least-squares planes of the two groups. In the crystal (Fig. 2), π–π overlap is observed for paired molecules with a centroid-centroid distance of *ca* 3.62 (1) Å between the benzene and pyrimidine rings of parallel 3-amino­quinazolin-4(3*H*)-one groups. N—H···N hydrogen bonds form R_6_^6^(30) rings and N—H···O form C(5) chains to generate three dimensional packing. Weak C—H···O contacts (C(5)) are also observed.

### Crystal data

**Table:**

C_11_H_13_N_3_O	*D*_x_ = 1.246 Mg m^−^^3^
*M**_r_* = 203.24	Cu *K*α radiation, λ = 1.54184 Å
Trigonal, *R*3:*H*	Cell parameters from 2040 reflections
*a* = 24.1525 (5) Å	θ = 5.0–74.1°
*c* = 9.6500 (2) Å	µ = 0.67 mm^−^^1^
*V* = 4875.1 (2) Å^3^	*T* = 296 K
*Z* = 18	Block, colourless
*F*(000) = 1944	0.34 × 0.25 × 0.19 mm

### Data collection

**Table:**

Agilent SuperNova Dual Source diffractometer with an Atlas detector	1913 reflections with *I* > 2σ(*I*)
ω scans	*R*_int_ = 0.013
Absorption correction: multi-scan (*CrysAlis PRO*; Agilent, 2014)	θ_max_ = 74.1°, θ_min_ = 3.7°
*T*_min_ = 0.975, *T*_max_ = 0.984	*h* = −21→30
3734 measured reflections	*k* = −26→18
2136 independent reflections	*l* = −11→10

### Refinement

**Table:**

Refinement on *F*^2^	Hydrogen site location: mixed
Least-squares matrix: full	H atoms treated by a mixture of independent and constrained refinement
*R*[*F*^2^ > 2σ(*F*^2^)] = 0.040	*w* = 1/[σ^2^(*F*_o_^2^) + (0.0637*P*)^2^ + 1.4157*P*] where *P* = (*F*_o_^2^ + 2*F*_c_^2^)/3
*wR*(*F*^2^) = 0.120	(Δ/σ)_max_ < 0.001
*S* = 1.06	Δρ_max_ = 0.17 e Å^−^^3^
2136 reflections	Δρ_min_ = −0.15 e Å^−^^3^
146 parameters	Extinction correction: *SHELXL2013* (Sheldrick, 2015), Fc^*^=kFc[1+0.001xFc^2^λ^3^/sin(2θ)]^-1/4^
0 restraints	Extinction coefficient: 0.00114 (10)

### Special details

**Table:**

Geometry. All e.s.d.'s (except the e.s.d. in the dihedral angle between two l.s. planes) are estimated using the full covariance matrix. The cell e.s.d.'s are taken into account individually in the estimation of e.s.d.'s in distances, angles and torsion angles; correlations between e.s.d.'s in cell parameters are only used when they are defined by crystal symmetry. An approximate (isotropic) treatment of cell e.s.d.'s is used for estimating e.s.d.'s involving l.s. planes.

### Fractional atomic coordinates and isotropic or equivalent isotropic displacement parameters (Å^2^)

**Table:**

	*x*	*y*	*z*	*U*_iso_*/*U*_eq_	
C1	0.23343 (6)	0.11120 (6)	0.00970 (12)	0.0453 (3)	
C2	0.28221 (6)	0.06090 (5)	0.14259 (13)	0.0457 (3)	
C3	0.27352 (5)	0.09197 (5)	0.26270 (12)	0.0440 (3)	
C4	0.24674 (6)	0.13113 (6)	0.24439 (12)	0.0450 (3)	
C5	0.29168 (6)	0.08293 (7)	0.39466 (14)	0.0535 (3)	
H5	0.3089	0.0563	0.4064	0.064*	
C6	0.28403 (7)	0.11345 (8)	0.50654 (14)	0.0618 (4)	
H6	0.2958	0.1074	0.5946	0.074*	
C7	0.25865 (8)	0.15367 (8)	0.48822 (14)	0.0630 (4)	
H7	0.2544	0.1749	0.5643	0.076*	
C8	0.23997 (7)	0.16241 (7)	0.36013 (14)	0.0567 (3)	
H8	0.2228	0.1891	0.3498	0.068*	
C9	0.20961 (7)	0.11882 (6)	−0.12831 (14)	0.0546 (3)	
H9A	0.2357	0.1157	−0.2009	0.066*	
H9B	0.2141	0.1610	−0.1337	0.066*	
C10	0.13977 (8)	0.06846 (8)	−0.15299 (18)	0.0697 (4)	
H10A	0.1359	0.0265	−0.1554	0.084*	
H10B	0.1141	0.0691	−0.0763	0.084*	
C11	0.11417 (10)	0.07946 (12)	−0.2869 (2)	0.0996 (7)	
H11A	0.1222	0.1226	−0.2894	0.149*	
H11B	0.0690	0.0503	−0.2924	0.149*	
H11C	0.1351	0.0724	−0.3639	0.149*	
N1	0.25905 (5)	0.07168 (5)	0.01918 (10)	0.0447 (3)	
N2	0.22710 (5)	0.14035 (5)	0.11608 (11)	0.0486 (3)	
N3	0.26483 (7)	0.04247 (6)	−0.10370 (12)	0.0550 (3)	
O1	0.30729 (5)	0.02780 (5)	0.14300 (11)	0.0625 (3)	
H3A	0.2287 (9)	0.0033 (10)	−0.1045 (18)	0.069 (5)*	
H3B	0.2972 (10)	0.0370 (9)	−0.087 (2)	0.069 (5)*	

### Atomic displacement parameters (Å^2^)

**Table:**

	*U*^11^	*U*^22^	*U*^33^	*U*^12^	*U*^13^	*U*^23^
C1	0.0447 (6)	0.0405 (6)	0.0464 (6)	0.0179 (5)	−0.0012 (5)	0.0016 (4)
C2	0.0425 (6)	0.0391 (5)	0.0518 (7)	0.0177 (5)	−0.0073 (5)	−0.0046 (5)
C3	0.0400 (6)	0.0401 (6)	0.0462 (6)	0.0158 (5)	−0.0028 (4)	−0.0002 (4)
C4	0.0441 (6)	0.0438 (6)	0.0439 (6)	0.0196 (5)	0.0021 (4)	0.0025 (4)
C5	0.0514 (7)	0.0554 (7)	0.0518 (7)	0.0253 (6)	−0.0067 (5)	0.0018 (5)
C6	0.0650 (8)	0.0724 (9)	0.0432 (7)	0.0307 (7)	−0.0043 (6)	0.0016 (6)
C7	0.0736 (9)	0.0695 (9)	0.0450 (7)	0.0350 (7)	0.0067 (6)	−0.0039 (6)
C8	0.0653 (8)	0.0603 (8)	0.0504 (7)	0.0358 (7)	0.0057 (6)	−0.0003 (6)
C9	0.0631 (8)	0.0512 (7)	0.0486 (7)	0.0279 (6)	−0.0060 (5)	0.0022 (5)
C10	0.0616 (9)	0.0732 (10)	0.0692 (9)	0.0300 (8)	−0.0086 (7)	0.0004 (7)
C11	0.0780 (12)	0.1036 (15)	0.0986 (15)	0.0315 (11)	−0.0333 (11)	0.0058 (12)
N1	0.0467 (5)	0.0401 (5)	0.0434 (5)	0.0189 (4)	−0.0036 (4)	−0.0052 (4)
N2	0.0546 (6)	0.0488 (6)	0.0462 (6)	0.0287 (5)	0.0001 (4)	0.0023 (4)
N3	0.0630 (7)	0.0499 (6)	0.0494 (6)	0.0263 (6)	−0.0042 (5)	−0.0120 (4)
O1	0.0729 (6)	0.0611 (6)	0.0683 (6)	0.0446 (5)	−0.0198 (5)	−0.0166 (4)

### Geometric parameters (Å, º)

**Table:**

C1—N2	1.2963 (16)	C7—H7	0.9300
C1—N1	1.3760 (16)	C8—H8	0.9300
C1—C9	1.4981 (17)	C9—C10	1.526 (2)
C2—O1	1.2209 (15)	C9—H9A	0.9700
C2—N1	1.3945 (16)	C9—H9B	0.9700
C2—C3	1.4520 (17)	C10—C11	1.512 (2)
C3—C4	1.3984 (18)	C10—H10A	0.9700
C3—C5	1.3991 (18)	C10—H10B	0.9700
C4—N2	1.3832 (16)	C11—H11A	0.9600
C4—C8	1.4032 (18)	C11—H11B	0.9600
C5—C6	1.371 (2)	C11—H11C	0.9600
C5—H5	0.9300	N1—N3	1.4219 (14)
C6—C7	1.395 (2)	N3—H3A	0.91 (2)
C6—H6	0.9300	N3—H3B	0.87 (2)
C7—C8	1.368 (2)		
			
N2—C1—N1	122.69 (11)	C1—C9—H9A	109.1
N2—C1—C9	118.73 (11)	C10—C9—H9A	109.1
N1—C1—C9	118.53 (11)	C1—C9—H9B	109.1
O1—C2—N1	120.11 (11)	C10—C9—H9B	109.1
O1—C2—C3	125.67 (12)	H9A—C9—H9B	107.9
N1—C2—C3	114.21 (10)	C11—C10—C9	112.30 (15)
C4—C3—C5	120.51 (12)	C11—C10—H10A	109.1
C4—C3—C2	118.94 (11)	C9—C10—H10A	109.1
C5—C3—C2	120.55 (11)	C11—C10—H10B	109.1
N2—C4—C3	122.27 (11)	C9—C10—H10B	109.1
N2—C4—C8	118.95 (12)	H10A—C10—H10B	107.9
C3—C4—C8	118.78 (12)	C10—C11—H11A	109.5
C6—C5—C3	119.73 (13)	C10—C11—H11B	109.5
C6—C5—H5	120.1	H11A—C11—H11B	109.5
C3—C5—H5	120.1	C10—C11—H11C	109.5
C5—C6—C7	119.93 (13)	H11A—C11—H11C	109.5
C5—C6—H6	120.0	H11B—C11—H11C	109.5
C7—C6—H6	120.0	C1—N1—C2	123.22 (10)
C8—C7—C6	121.04 (13)	C1—N1—N3	118.60 (10)
C8—C7—H7	119.5	C2—N1—N3	118.13 (10)
C6—C7—H7	119.5	C1—N2—C4	118.58 (11)
C7—C8—C4	119.99 (13)	N1—N3—H3A	104.0 (11)
C7—C8—H8	120.0	N1—N3—H3B	103.8 (13)
C4—C8—H8	120.0	H3A—N3—H3B	108.1 (17)
C1—C9—C10	112.34 (12)		
			
O1—C2—C3—C4	176.78 (12)	N2—C1—C9—C10	−89.31 (15)
N1—C2—C3—C4	−3.10 (16)	N1—C1—C9—C10	88.39 (15)
O1—C2—C3—C5	−3.2 (2)	C1—C9—C10—C11	175.23 (16)
N1—C2—C3—C5	176.95 (11)	N2—C1—N1—C2	−1.99 (18)
C5—C3—C4—N2	−178.79 (11)	C9—C1—N1—C2	−179.59 (11)
C2—C3—C4—N2	1.26 (17)	N2—C1—N1—N3	−179.17 (11)
C5—C3—C4—C8	1.61 (18)	C9—C1—N1—N3	3.23 (16)
C2—C3—C4—C8	−178.34 (11)	O1—C2—N1—C1	−176.35 (11)
C4—C3—C5—C6	−0.98 (19)	C3—C2—N1—C1	3.54 (16)
C2—C3—C5—C6	178.97 (12)	O1—C2—N1—N3	0.84 (17)
C3—C5—C6—C7	−0.4 (2)	C3—C2—N1—N3	−179.27 (10)
C5—C6—C7—C8	1.2 (2)	N1—C1—N2—C4	−0.22 (18)
C6—C7—C8—C4	−0.6 (2)	C9—C1—N2—C4	177.38 (11)
N2—C4—C8—C7	179.55 (13)	C3—C4—N2—C1	0.51 (18)
C3—C4—C8—C7	−0.8 (2)	C8—C4—N2—C1	−179.89 (12)

### Hydrogen-bond geometry (Å, º)

**Table:**

*D*—H···*A*	*D*—H	H···*A*	*D*···*A*	*D*—H···*A*
N3—H3*A*···N2^i^	0.91 (2)	2.16 (2)	3.0677 (17)	176.1 (16)
N3—H3*B*···O1^ii^	0.87 (2)	2.51 (2)	3.0599 (16)	122.0 (15)
C5—H5···O1^iii^	0.93	2.44	3.3163 (16)	157

Symmetry codes: (i) *y*, −*x*+*y*, −*z*; (ii) −*y*+1/3, *x*−*y*−1/3, *z*−1/3; (iii) −*x*+*y*+2/3, −*x*+1/3, *z*+1/3.
