# Supplementary figures and images for: Crystal structure of 3-amino-2-propyl­quinazolin-4(3H)-one
Source: Acta Crystallogr E Crystallogr Commun. 2015 Jul 22;71(Pt 8):o590–1. doi: 10.1107/S2056989015013134 (PMC4571413; doi:10.1107/S2056989015013134)

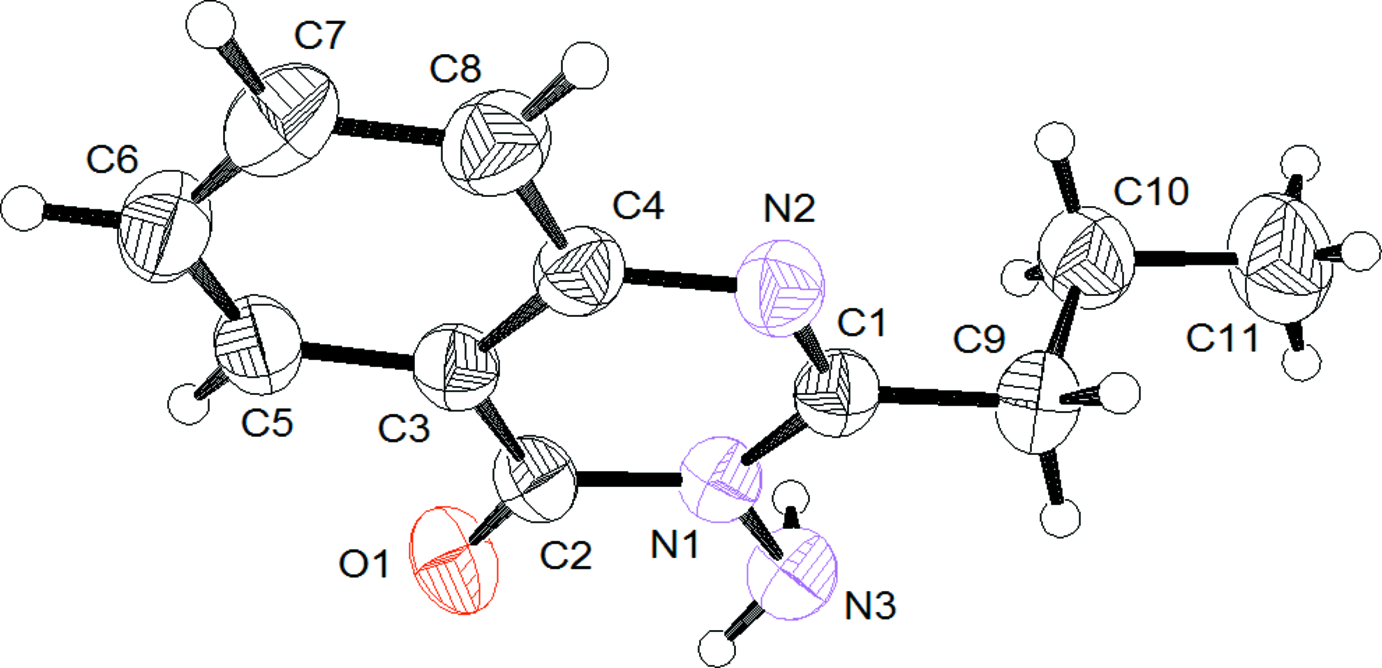

Supplement: Supplementary file 4 [file e-71-0o590-fig1.tif]

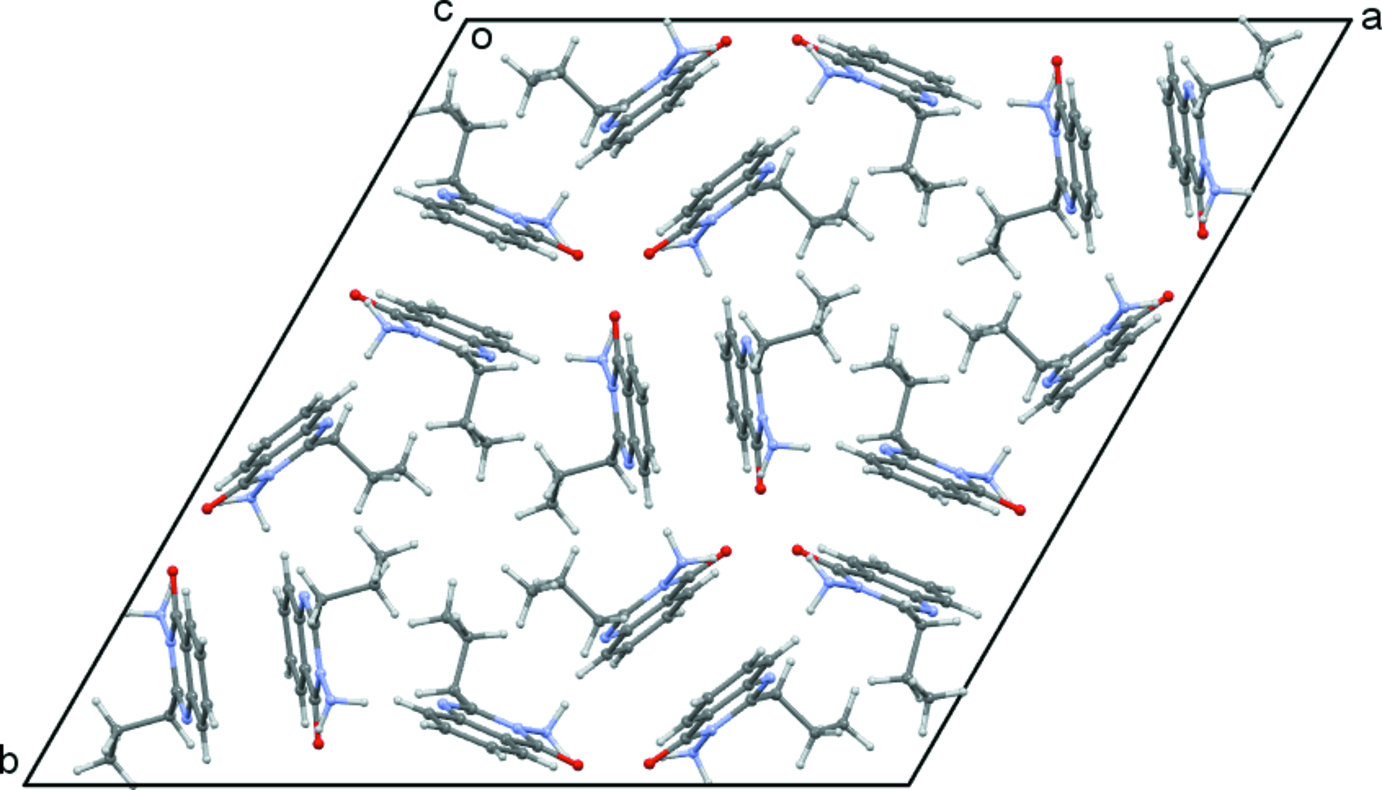

Supplement: Supplementary file 5 [file e-71-0o590-fig2.tif]
